# Publication Times in Ophthalmology Journals: The Story of Accepted Manuscripts

**DOI:** 10.7759/cureus.17738

**Published:** 2021-09-05

**Authors:** Tomasz Skrzypczak, Jakub Michałowicz, Marta Hossa, Michał Mamak, Aleksandra Jany, Anna Skrzypczak, Joanna Bogusławska, Agnieszka Kowal-Lange

**Affiliations:** 1 Medicine, Wroclaw Medical University, Wroclaw, POL; 2 Dentistry, Wroclaw Medical University, Wroclaw, POL; 3 Ophthalmology, Provincial Specialist Hospital in Wroclaw, Research and Development Center, Wroclaw, POL

**Keywords:** publication times, ophthalmology journals, peer-review, articles, scientific papers, research and publication, journal

## Abstract

Introduction: The process of scientific publishing changed greatly in the past decades. The authors aimed to get insight into the time required for articles to be accepted and released online in high-impacted ophthalmology journals.

Methods: Comprehensive review of all original articles published by eight ophthalmology journals during a one-year period was performed for 2020 and 2005. Time taken from submission to acceptance and the first online release of the article was abstracted and analyzed.

Results: A total of 3110 articles were reviewed. In 2020, the overall median time from submission to acceptance (AT) was 119 days (IQR 83-168) and 30 days (10-71) from acceptance to the first online release of the article (OP). AT increased by 7.3% from 2005 to 2020, whereas OP reduced by 73%. Publications, which the corresponding author was affiliated with US-located institution had shorter both AT and OP in 2005 and 2020. The author’s specialty in ophthalmology had an inconclusive impact on AT and OP. Papers with multiple affiliated institutions had shorter AT and OP in both 2005 and 2020; however, these differences were not statistically significant.

Conclusion: This study demonstrated that increasing pressure on authors, editors, and reviewers to publish articles and journals with high impact factor (IF) significantly influenced publication times in ophthalmology journals. Inflation of research papers was associated with rising AT time. A significant decrease in OP time was potentially explained by the editor’s demand to achieve decent journal IF. This article brings to light relative publication times in the ophthalmology scientific journals.

## Introduction

Scientific publication in established medical journals disseminates research results within the professional community [1]. The authors of the article contribute to the advancement of their disciplines and clinical practice [1]. This remains true across all medical specialties [1]. Ophthalmology is no exception.

The process of the scientific publication changed greatly in the past decades. The well-known “publish or perish” dilemma highlights the main incentive fueling the need to publish as much as possible [2]. Authors strive to publish in journals with high impact factor (IF), which became the superior method of academic evaluation in the contemporary scientific world [2,3]. Those who fail in this race may miss career opportunities, research funding, or other rewards [2,4,5]. Publication inflation leads to fierce competition not only between authors but also between editors. As the consequence, many papers are published after being rejected several times by other journals [4,6]. In an effort to publish, the time dedicated to formatting and then submitting dramatically increased, as each journal has different specifications and requirements for submission [6]. It seems reasonable that also journals struggle to handle a rising wave of submitted manuscripts.

The long time between invention and the introduction of significant breakthroughs has a negative impact on the patients seeking new therapeutic options [1,7,8]. Delays block authors from getting recognition for their publication and journals from collecting citations [1,9-11]. It was demonstrated that speed of review/publication process, journal quality, and topic fit are the most significant factors that influence an author’s choice of a journal [1,12]. As a result, long publication times might impact an author’s decision to publish in a specific journal [1,9]. Short publication times could potentially benefit the scientific community, although not at the cost of articles quality [1,13].

The idea of publication delay traditionally refers to the time between the acceptance of an article and in-print publication [14]. The introduction of online access established publishing of initial form of articles before formal in-print release a standard in today’s research world [14]. In most contemporary scientific journals, publication delay is a period between submission and the date of the first online release of the article. The paper has an extra time of visibility prior to formal in-print publication and can get more citations. This tactic was found to increase the IF of ophthalmology journals [9]. However, exact publication times from submission to the first online release of the article in ophthalmology journals were not previously revealed.

A bibliometric analysis is useful to understand and analyze various aspects of the scientific publication process in selected disciplines [1,11,15]. To date, there was little data concerning how turnover time from submission to acceptance has changed through the years, in ophthalmology journals. The purpose of this study was to evaluate the time required for articles to be accepted and released online in ophthalmology journals in 2020. The authors aimed to determine, whether these times have changed since 2005.

## Materials and methods

Journal selection 

First, all 60 journals under the “Ophthalmology” category in the Journal Citation Report 2019® database were considered. Authors excluded journals publishing mainly the systematic reviews, because most of these are invited or solicited and, therefore, do not strictly follow a timeline [9]. Journals that are focused on related to ophthalmology but very different disciplines such as molecular biology, neuroscience, and optometry were excluded from the analysis. Neuro-ophthalmology journals, which encompass both ophthalmology and neurology were not analyzed. These made the analysis oriented only to studies in clinical and experimental ophthalmology. To date, there was no established definition of what IF value makes a journal high impacted [3]. In this study, selected ophthalmology journals were dichotomized by median IF. It was assumed that publication times in high-impact-factor journals are more important to authors, thus only journals with IF ≥ median were included in the analysis. Then the journals were assessed for dates of submission, acceptance, and first online release of articles in 2020 and 2005. Journals that did not provide these data on publications were excluded from the study. Finally, the following eight ophthalmology journals were analyzed: Ophthalmology (IF 8.470), Investigative Ophthalmology & Visual Science (IOVS, IF 3.470), Acta Ophthalmologica (Acta Ophthalmol., IF 3.362), Experimental Eye Research (Exp. Eye Res., IF 3.011), Graefe’s Archive for Clinical and Experimental Ophthalmology (Graefe’s Arch. Clin. Exp. Ophthalmol., IF 2.396), Ophthalmic Research(Ophthalmic Res., IF 1.961), Ophthalmologica (IF 1.926), and Current Eye Research (Curr. Eye Res., IF 1.754).

**Figure 1:**
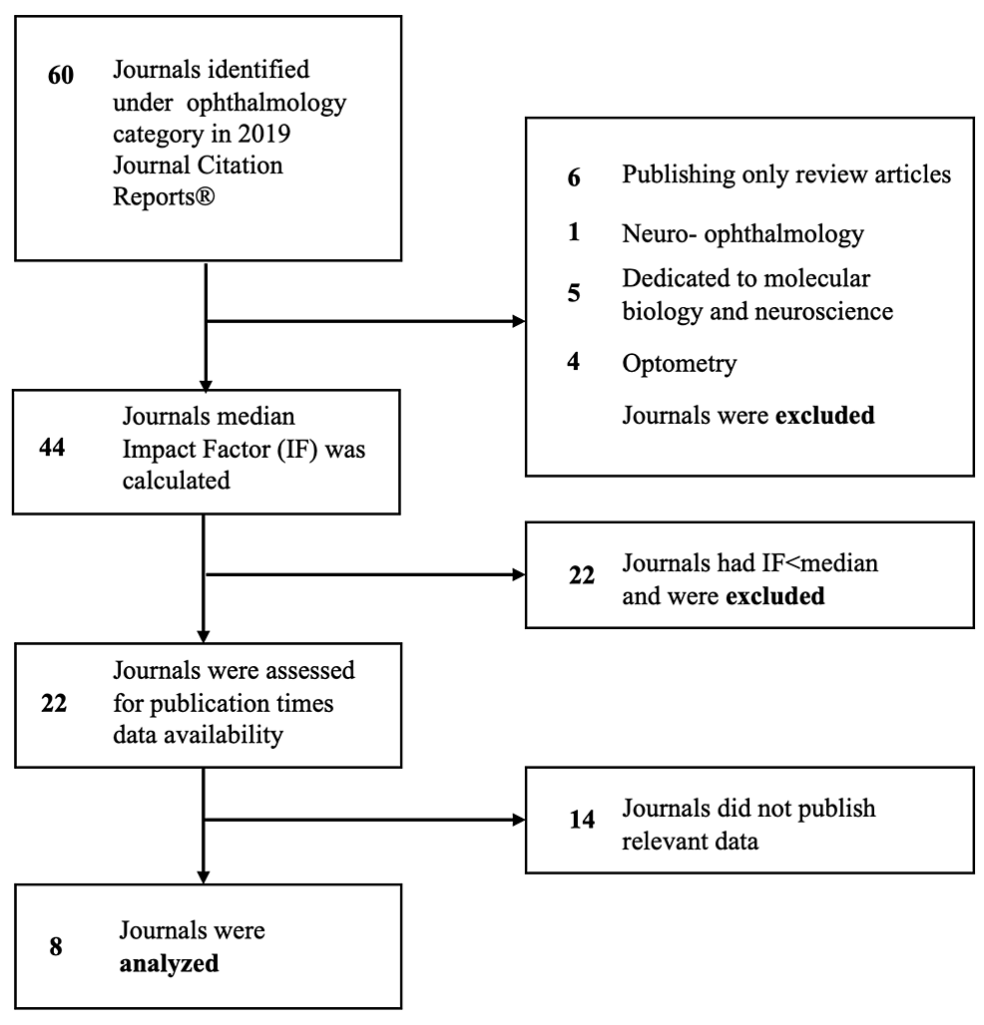
Flow diagram of ophthalmology journals selection process.

Figure 1 presented the journal selection process.

Data extraction

A comprehensive review of all original articles published by these journals was conducted for 2005 and 2020 during a one-year period (January through December). The authors obtained data only from original articles. All values were double assessed independently by two researchers. Articles with missing values were excluded from the analysis. Articles classified as book reviews, correspondence, expert opinion, continuing medical education, letters, discussions, literature review, commentaries, editorials, tips, innovations, ideas, and case reports were excluded from the study [1]. The date of submission, time of acceptance, and first online release of the publication time were extracted from the included articles. The number of institutions and the country of the corresponding author was collected. The articles were categorized according to their study design into one of the following groups: basic science, databases, cross-sectional, case-series, case-control, retrospective cohort, prospective cohort, randomized trial, and others [1]. To determine the primary specialty of the authors in selected journals, it was extracted whether the authors were ophthalmologists or were affiliated with other departments. These were classified as (1) all authors were ophthalmologists or affiliated with ophthalmology departments/divisions (AO); (2) at least one author was an ophthalmologist/affiliated with ophthalmology department/divisions and one author was not (PO); or (3) none of the authors was an ophthalmologist or affiliated with ophthalmology department/division (NO).

Results analysis

Acceptance time (AT) was defined as the number of days from submission date to acceptance date [1]. Online publication time (OP) was defined as the number of days from the date of acceptance to the date of the first online release of the article. Categorical variables were summarized as percentages and proportions [1]. Continuous data were presented as the median and interquartile range (IQR) for the 25th to 75th percentile [1]. The normality of the data was examined with the Shapiro-Wilk test. Data were analyzed with Mann-Whitney U test, student’s t-test, and Kruskal-Wallis tests. P<0.05 was considered statistically significant. Statistical analyses were performed with open-source software JASP, version 0.14.1 (https://jasp-stats.org). Data were collected with the use of Microsoft Excel software, version 16.48, (Microsoft Corporation, Redmond, WA, USA).

## Results

Overall, data were extracted from 3,110 articles, 1,528 in 2005, and 1,582 were published in 2020. In 2005, there were 79 issues in total, whereas 82 issues were published in 2020. Analyzed articles published by IOVS were the most common both in 2005 and 2020 (628; 41% and 379; 24% of annual overall volume, respectively). The median IF used to dichotomize journals in the selection process was 1.754. Table 1 summarized journal characteristics.

**Table 1:** Journal characteristics and articles volume. Exp. Eye Res.: Experimental Eye Research; Graefe’s Arch. Clin. Exp. Ophthalmol.: Graefe’s Archive for Clinical and Experimental Ophthalmology; IOVS: Investigative Ophthalmology & Visual Science

Journal	Impact factor	Number of issues			Number of original articles, N(%)	
		2020	2005	2020(%)		2005(%)
Acta Ophthalmologica	3.362	8	6	235(15%)		101(7%)
Current Eye Research	1.754	12	12	175(11%)		122(8%)
Exp. Eye Res.	3.011	12	12	269(17%)		156(10%)
Graefe’s Arch. Clin. Exp. Ophthalmol.	2.396	12	12	287(18%)		141(9%)
IOVS	3.470	14	13	379(24%)		628(41%)
Ophthalmic Research	1.961	6	6	61(4%)		46(3%)
Ophthalmologica	1.962	6	6	55(3%)		66(4%)
Ophthalmology	8.470	12	12	121(8%)		268(18%)
Overall		82	79	1,582		1,528

Acceptance time

The AT times in 2020 for each study design were split by country, the specialty of the authors, and the number of institutions that participated in the study (Table 2).

**Table 2:** Time from submission to acceptance in 2020 (days). AT: Time from submission to acceptance; AO: all authors were ophthalmologists or affiliated with ophthalmology departments/divisions; PO: at least one author was an ophthalmologist/affiliated with ophthalmology department/divisions; and one author was not; NO: none of the authors was an ophthalmologist or affiliated with ophthalmology department/division.

AT time, median (IQR)	Basic Science	Case Series	Case - control	Cross - sectional	Databases	Prospective cohort	Retrospective cohort	Randomized Trial	Other	Overall
Country										
US	107	103	111	92	71	106	81	175	-	106
	(72-146)	(97-119)	(73-129)	(70-132)	(46-108)	(99-172)	(75-152)	(93-166)	-	(74-149)
Non-US	128	108	115	54	134	139	211	178	127	122
	(91-174)	(78-163)	(80-165)	(86-174)	(75-141)	(84-177)	(82-175)	(90-181)	(115-153)	(84-173)
Affiliation										
Study from AO	96	69	408	75	152	131	106	282	135	117
	(89-165)	(82-159)	(86-184)	(80-160)	(57-111)	(79-177)	(77-159)	(80-173)	(129-141)	(82-166)
Study from PO	158	120	70	98	134	177	177	115	118	122
	(86-169)	(80-158)	(70-151)	(88-173)	(79-139)	(95-180)	(94-190)	(108-182)	(124-160)	(88-174)
Study from NO	107	75	74	131	112	125	157	113	193	113
	(80-154)	(75-75)	(81-131)	(58-202)	(51-142)	(86-130)	(84-129)	(58-145)	(193-193)	(75-157)
Number of institutions										
Greater or equal to median	125	69	70	75	152	155	176	139	127	117
	(87-162)	(83-152)	(80-155)	(78-155)	(57-130)	(82-173)	(80-169)	(79-172)	(138-180)	(81-164)
Less than median	158	164	200	81	165	165	177	104	121	123
	(83-171)	(78-161)	(73-169)	(96-188)	(75-140)	(92-177)	(90-182)	(102-179)	(121-130)	(88-176)
Overall										
	121	107	112	118	110	120	111	136	142	119
	(86-166)	(79-158)	(79-157)	(83-170)	(61-138)	(85-177)	(82-172)	(92-179)	(125-167)	(83-168)

Overall median AT time increased from 111 (77-152) days in 2005 to 119 (83-168) days in 2020; (7.3%), P<.001. Publications whose corresponding author was affiliated with US institution had significantly shorter AT, 106 (74-149) days than others, 122 (84-173) days, P<.001. This trend was also revealed for articles published in 2005; 122 (84-177) and 135 (90-194) days, respectively, P=0.003. In 2020, NO author’s publications had the shortest AT time 113 (75-157) days in contrast to the PO author category, which waited the longest time to get their articles accepted; 122 (82-174) days; P=0.041. On the other hand, NO author group had the longest and PO had the shortest AT time in 2005; 143 (92-212) and 124 (84-183) days, respectively; P=0.020. In 2020, the median number of institutions affiliated with authors, in analyzed articles was 3 (2-4) and was higher than in 2005, 2(1-3). Papers with ≥median affiliated institutions had shorter AT times both in 2020 and 2005 than these with < median number of institutions; however, these differences were not statistically significant; 117 (81-164) and 123 (88-176) days, P=0.163 and 129 (87-188) and 131 (90-186), P=0.979, respectively.

Online publication time

The OP times in 2020 for each study design were split by country, the specialty of the authors, and the number of institutions that participated in the study (Table 3).

**Table 3:** Time from acceptance to first online release of article in 2020 (days). OP: Time from acceptance to first online release of publication; AO: all authors were ophthalmologists or affiliated with ophthalmology departments/divisions; PO: at least one author was an ophthalmologist/affiliated with ophthalmology department/divisions; and one author was not; NO: none of the authors was an ophthalmologist or affiliated with ophthalmology department/division.

OP time, median (IQR)	Basic Science	Case Series	Case - control	Cross - sectional	Databases	Prospective cohort	Retrospective cohort	Randomized Trial	Other	Overall
Country										
US	25	14	34	19	70	40	55	49	-	21
	(8-50)	(10-52)	(9-42)	(8-36)	(9-78)	(15-70)	(8-36)	(7-9)	-	(8-48)
Non-US	23	31	45	35	49	57	39	54	132	32
	(7-55)	(11-63)	(16-165)	(13-70)	(33-208)	(15-197)	(13-181)	(10-221)	(209-269)	(11-79)
Affiliation										
Study from AO	40	21	50	35	144	50	54	26	227	27
	(6-50)	(12-60)	(11-65)	(13-63)	(51-241)	(15-103)	(11-63)	(7-184)	(206-247)	(11-67)
Study from PO	34	37	22	36	31	67	65	72	159	33
	(8-56)	(10-66)	(21-209)	(10-66)	(28-208)	(26-219)	(10-206)	(8-47)	(164-241)	(10-80)
Study from NO	30	1	44	35	34	46	92	142	272	29
	(5-41)	-	(27-59)	(28-44)	(32-56)	(28-52)	(9-193)	(25-52)	-	(8-50)
Number of institutions										
Greater or equal to median	28	21	22	35	49	61	51	72	132	29
	(7-47)	(12-54)	(15-66)	(11-54)	(41-176)	(15-172)	(12-81)	(8-125)	(148-240)	(11-64)
Less than median	34	54	45	29	145	45	65	26	246	33
	(8-64)	(9-68)	(16-212)	(13-71)	(22-203)	(15-197)	(9-194)	(7-41)	(238-260)	(9-83)
Overall										
	23	26	41	29	60	48	31	11	232	30
	(7-51)	(11-61)	(15-96)	(12-60)	(28-208)	(15-186)	(11-104)	(8-57)	(191-249	(10-71)

Overall mean OP time decreased from 111 (77-152) days in 2005 to 30 (10-71) days in 2020; (73%), P<.001. In 2020 and 2005 publications, in which the corresponding author was affiliated with a US-based institution had shorter OP times 21 (8-48) and 103 (66-131) days than those which the corresponding author was not affiliated with any of the US located institutions; 116 (83-174) and 32 (11-79) days, respectively, all P<.001. In 2020, AO author’s publications had the shortest OP time 27 (11-67) days in contrast to the PO author category, which waited the longest time to get their articles accepted; 33 (10-80) days; P=0.007. In contrast, the AO author group had the longest OP time, 114 (82-160) days and authors classified into the NO group waited for the shortest for the first release of their articles, 107 (66-150) days in 2005. However, these differences were not statistically significant P=0.148. Papers with ≥median affiliated institutions had shorter OP times both in 2020 and 2005 than these with < median number of institutions; however, this difference was only statistically significant in 2005; 29 (11-64) and 33 (9-83) days, P=0.405 and 109 (72-148) and 117 (87-169), P<.001, respectively.

## Discussion

The results showed that in 2020 the median time from submission to acceptance in eight analyzed ophthalmology journals was 119 days, with a fast turnaround time from acceptance to the first online release of the article (median of 30 days). Overall submission to AT was longer (7%) in 2020 than in 2005. Time from acceptance to the first online release of the article significantly declined from 111 days to 30 days (73%) in 2020. Only slight growth in the number of published articles (3.5%) and journal volumes (3.8%) accompanied these differences. Several reasons could potentially explain these findings.

For the past two centuries, the volume of peer-reviewed articles published per year has increased by a relatively steady 3.5% per year [16]. The research community continues to see peer review as fundamental to scholarly communication and appears committed to it despite some perceived shortcomings. The typical reviewer spends five hours for peer review and reviews eight articles a year. Peer review is under pressure, notably from growth in research outputs [16,17]. On the other hand, many publishers and editors see it as their duty to implement editorial policies that will increase the journal IF [4]. Journal editors potentially avoid publishing specific article types such as case reports [3]. Publications in topics with narrow public interest or manuscripts with negative findings are less appealing for citations [3]. This discrimination might inflate journal IF [3]. As a consequence, the rate of submissions, subscriptions, and advertisements would definitely increase, which is desired by the journal’s administrators [3,18]. In addition, IF measures the average citations per journal [3]. Subsequently, IF awards limited productivity and punishes extra output [3]. Cumulation of these factors leads to increasing conflict between editors, reviewers, and researchers that are under pressure to publish highly impacted journals and articles. This fierce competition is a reasonable explanation for longer periods between submission and acceptance of the articles in ophthalmology journals.

With the breakneck development of online technology and prevalent internet access, traditional in-print publications are no longer crucial for articles to get located in the scientific literature [1]. Rapid online access to preliminary and final versions of accepted articles made research dissemination faster and significantly decreased publication times [1,9,19,20]. In analyzed articles, OP was significantly shorter in 2020 than in 2005. This translates to increased article accessibility and rises in the IF of a journal, which is important for both authors and editors [1,9,14,19,20].

It is rational that medical researchers aim to publish articles in highly cited journals [21]. On the other hand, journal editors strive to select the most credible, important, and novel research [21]. However, this selection process is often impacted by the environment in which they work [21]. The corresponding author’s country of residence, eloquence and English language fluency of the authors were associated with the higher number of citations and greater chances of being accepted in top-ranked medical journals [21-23]. This coincides with our study findings. Publications in which the corresponding author originated from US located institutions had significantly shorter times from submission to acceptance and from acceptance to the first online release of the article.

Based on the presented results, it was impossible to conclude whether the author’s specialty influenced articles publication times. The previous investigation demonstrated that authors' affiliation had an impact on research productivity [24]. Other studies revealed no or weak associations with the publication times and the author’s specialty [1,25].

Local, regional, and national collaboration increase research productivity by expanding the availability of resources and connections between institutions and their researchers [26]. Articles with more coauthors had better quality and were more likely to be cited [27]. The authors found that both in 2020 and 2005, articles with a greater number of coauthors had significantly shorter publication times. However, this difference was only statistically significant for OP in 2005.

Although this investigation included a complex bibliometric analysis of more than 3,000 articles in respected journals in ophthalmology, there are some limitations. Only, data from eight ophthalmology journals in ophthalmology in two years (2020 and 2005) were included without comparison to journals in other disciplines. 2005 and 2020 were selected arbitrarily by the authors. The total number of submitted articles varied between included journals and might affect the results. Reporting the dates of publication is optional and this led to journal selection bias. Peer-review methodologies varied between different types of articles. The reported data could be affected by the journal composition of specific types of articles. According to these limitations, future research should include more journals and encompass broad spectrum ophthalmology subspecialties. Analysis of several consecutive years could ensure more reliable results. The authors were unable to evaluate whether observed changes had a tendency or were constant in time.

## Conclusions

In 2020, the median time from submission to acceptance was 119 days with fast turnaround times from acceptance to the first online release of the article (30 days). From 2005 to 2020, there was a 7.3% increase in time from submission to acceptance, whereas the time from acceptance to the first online release of the article was significantly reduced (by 73%). Publications whose corresponding author was affiliated with US-located institutions had significantly shorter publication times both in 2005 and 2020. The author’s specialty inconsistently influenced publication times in 2005 and 2020. This article presents relative publication speeds, so authors can make more informed decisions during submission.
